# Broadly neutralizing antibodies for HIV-1: efficacies, challenges and opportunities

**DOI:** 10.1080/22221751.2020.1713707

**Published:** 2020-01-27

**Authors:** Yubin Liu, Wei Cao, Ming Sun, Taisheng Li

**Affiliations:** a Department of Infectious Diseases, Peking Union Medical College Hospital, Peking Union Medical College and Chinese Academy of Medical Sciences, Beijing, People’s Republic of China; b Institute of Medical Biology, Chinese Academy of Medical Sciences, Kunming, People’s Republic of China; c Yunnan Key Laboratory of Vaccine Research & Development on Severe Infectious Diseases, Kunming, People’s Republic of China; d Clinical Immunology Center, Chinese Academy of Medical Sciences, Beijing, People’s Republic of China; e Tsinghua University Medical College, Beijing, People’s Republic of China

**Keywords:** HIV-1, broadly neutralizing antibodies (bNAbs), efficacies, challenges, opportunities

## Abstract

Combination antiretroviral therapy (cART) is effective but not curative, and no successful vaccine is currently available for human immunodeficiency virus-1 (HIV-1). Broadly neutralizing antibodies (bNAbs) provide a new approach to HIV-1 prevention and treatment, and these promising candidates advancing into clinical trials have shown certain efficacies in infected individuals. In addition, bNAbs have the potential to kill HIV-1-infected cells and to affect the course of HIV-1 infection by directly engaging host immunity. Nonetheless, challenges accompany the use of bNAbs, including transient suppression of viraemia, frequent emergence of resistant viruses in rebound viraemia, suboptimal efficacy in virus cell-to-cell transmission, and unclear effects on the cell-associated HIV-1 reservoir. In this review, we discuss opportunities and potential strategies to address current challenges to promote the future use of immunotherapy regimens.

## Introduction

Human immunodeficiency virus-1 (HIV-1) is the causative agent of acquired immunodeficiency syndrome (AIDS) and mainly infects CD4-positive (CD4^+^) immune cells, progressively damaging the immune system [1]. Without defence and surveillance by the immune system, individuals with HIV-1 infection are more vulnerable to pathogenic microorganisms and gene mutations, resulting in opportunistic infections and cancers and even death. Regardless, there currently are no effective HIV-1 vaccines and little hope for protective immunization. In a naturally infective course, the average survival time of patients is approximately 10 years. However, the introduction of combination antiretroviral therapy (cART) as a breakthrough has altered the course trajectory. Indeed, cART can dramatically increase the life expectancy of infected individuals by suppressing viral replication, promoting immune reconstitution, and preventing the onset of AIDS; in addition, cART might decrease the number of new infections when administered as part of pre- or postexposure prophylaxis [2–4]. Despite suppression of plasma viraemia, cART is not curative because these drugs fail to eliminate the latent HIV-1 reservoir [5], and the suppressed virus rebounds quickly in the vast majority of HIV-1-infected individuals when treatment is discontinued. As a result, daily and lifelong therapy is required, with numerous side effects.

The recent development of HIV-1-specific potent broadly neutralizing antibodies (bNAbs) provides a new approach for preventing, treating, and potentially even eradicating HIV-1 infection. Due to their important features, including a longer half-life, excellent safety and engaging the host immune response, bNAbs are being strongly pursued and developed [6, 7]. Functions, such as neutralizing free viruses, clearing infected cells and inhibiting cell-to-cell transmission of HIV-1, have been reported in a variety of studies [8–10]. Moreover, the growing profile of bNAbs provides new insight for rational vaccine design and promising immunogen testing [11].

## The development of broadly neutralizing antibodies

**Figure 1.**
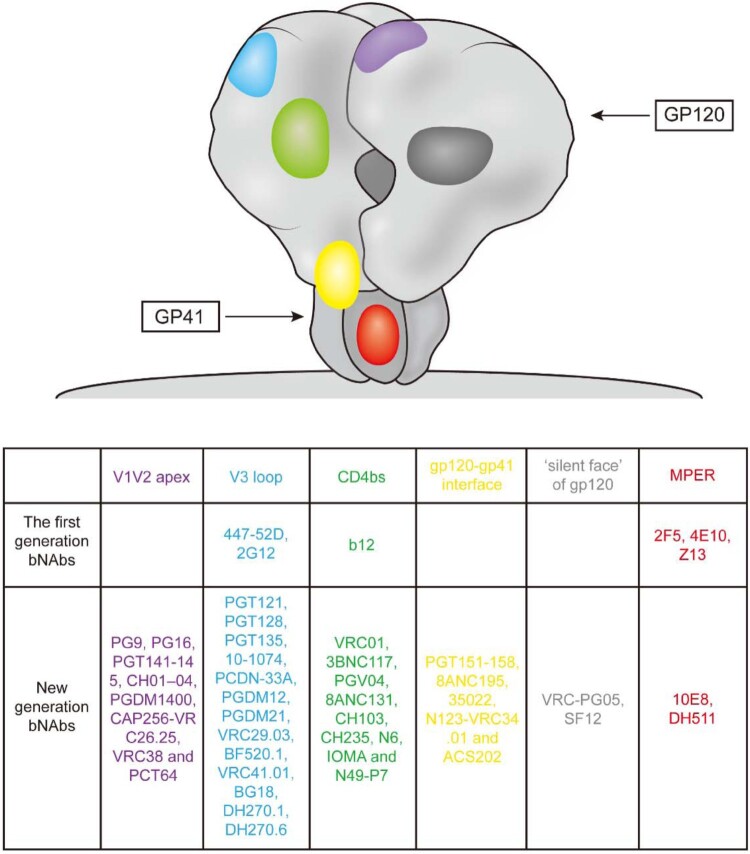
Binding sites for broadly neutralizing antibodies on HIV-1 envelope. Colors indicate approximate locations of six sites on surface representation (each site is shown once per trimer): V1V2 apex (purple), V3 loop (blue), CD4-binding site (CD4bs, green), gp120-gp41 interface (yellow), “silent face” of gp120 (dark gray), and the membrane proximal external region (MPER, red). mAbs are shown that recognize each site.

First-generation bNAbs were isolated in the early 1990s, mainly by using phage display and human hybridoma electrofusion. These bNAbs included b12, 447-52D, 2G12, 17b, 2F5, 4E10 and Z13, with different specificities [12–15] (Figure 1). Although these bNAbs neutralized diverse primary strains of HIV-1 in vitro, their potency and breadth were less than ideal [16]. For example, clinical trials showed that during interruption of cART, the combination of three neutralizing antibodies (2G12, 2F5 and 4E10) only moderately suppressed viraemia both in acutely and chronically HIV-1-infected individuals. Furthermore, the emergence of variants resistant to 2G12 was observed in most (12/14 and 7/8) of the recipients, and these escape mutants developed very rapidly and showed high titres [17, 18]. In addition, 2F5 and 4E10 are self-reactive [19, 20].

Although early studies with these first-generation bNAbs showed less than optimal results, the development of high-throughput neutralization assays and single-cell antibody cloning techniques have facilitated the isolation and characterization of a new generation of bNAbs with much greater potency and breadth for immune prophylaxis and therapy of HIV-1. These potent bNAbs were isolated by single-B-cell culture and direct functional screening or antigen-specific single-B-cell sorting. Both methods identified multiple bNAbs and new HIV-1 spike sites of vulnerability to these neutralizing antibodies [21] (Figure 1). This new generation of bNAbs displayed a 10- to 100-fold increase in potency and a more than 2-fold improvement in coverage than the earlier bNAbs. In addition to their strong activity in vitro, these new agents showed encouraging effects for both therapy and prevention in vivo. For example, studies in rhesus macaques showed that passive administration of bNAbs can protect against a high-dose viral challenge or repeated low-dose challenges at significantly lower serum concentrations [22, 23]. In immunotherapy experiments, administration of bNAb to chronically infected animals resulted in a rapid decline of plasma viral RNA to undetectable levels and a reduction in proviral DNA in the peripheral blood, gastrointestinal mucosa and lymph nodes. Moreover, host Gag-specific T cell responses were enhanced with monoclonal antibody treatment [24].

## Efficacy of next-generation bNAbs in clinical trials

With the encouraging results of preclinical studies, certain candidates from the new generation of bNAbs have been evaluated in clinical trials for the prevention and treatment of HIV-1 infection. To date, antibodies against the CD4 binding site, VRC01, 3BNC117, VRC01-LS and VRC07-523LS, as well as antibodies targeting the glycan-rich V3 loop, 10–1074 and PGT121, have been reported in humans (Table 1).

**Table 1.** Efficacy of next-generation bNAbs in clinical trials.

bNAbs	Epitope	Strategies	Note
VRC01	CD4bs	HIV-negative healthy adults: 20 mg/kg, i.v. (0w) 5, 20, and 40 mg/kg, i.v. (0w, 4w) 5 mg/kg, s.c. (0w, 4w)	Half-life: 15 days
		HIV-exposed infants: 20 or 40 mg/kg, s.c. (0w) 40 (0w) + 20 mg/kg monthly (6–18 times)	Half-life may change with repeat administration or increasing age
		On viremic subjects: 40 mg/kg, i.v. (0w)	Half-life: 12 days 40 mg/kg: 1.1- 1.8 log10 drop in 6/8, nadir on day9
		bNAbs during ATI: A5340 trial: 40 mg/kg, i.v. (−1w, 2w, 5w) NIH trial: 40 mg/kg, i.v. (−3d, 2w, 4w, 8w, 3, 4, 5, 6 m)	Delay in viral rebound: 4 weeks in the A5340 trial 5.6 weeks in the NIH trial
		bNAbs during ATI in adults with acutely treated HIV: 40 mg/kg, i.v. (0w, 3w, 6w, … , 24w)	Delay in viral rebound: does not lead to prolonged viral suppression
3BNC117	CD4bs	HIV-negative healthy adults: 1, 3, 10, or 30 mg/kg, i.v. (0w)	Half-life: 17 days
		On viremic subjects: 1, 3, 10 or 30 mg/kg, i.v. (0w)	Half-life: 9 days 30 mg/kg: 1.48 (0.8–2.5) log10 drop, nadir on day7
		bNAbs during ATI: 30 mg/kg, i.v. (0w, 3w) 30 mg/kg, i.v. (0w, 2w, 4w, 6w)	Delay in viral rebound: Two infusion: 6.7w (5–9w) Four infusion: 9.9 w (3–19w)
10–1074	V3 glycan	HIV-negative healthy adults: 3, 10, or 30 mg/kg, i.v. (0w)	Half-life: 24 days
		On viremic subjects: 3, 10 or 30 mg/kg, i.v. (0w)	Half-life: 12.8 days 30 mg/kg:1.52 (0.9–2.06) log10 copies/ml drop in 11/13; nadir on day10.3
3BNC117+10-1074	CD4bs+ V3 glycan	On viremic subjects: 30 mg/kg, i.v. (0w) 30 mg/kg, i.v. (0w, 2w, 4w)	30 mg/kg: 2.05 log10 copies/ml drop in 4/7
		bNAbs during ATI: 30 mg/kg, i.v. (0w, 3w, 6w)	Delay in viral rebound: 21w (5–30w)
VRC01-LS	CD4bs	HIV-negative healthy adults: 5, 20, or 40 mg/kg, i.v. (0w) 5 mg/kg, s.c. (0w) 20, and 40 mg/kg, i.v. (0w, 12w, 24w) 5 mg/kg, s.c. (0w, 12w, 24w)	Half-life: 71 ± 18 days (i.v.) 66 ± 24 days (s.c.)
VRC07-523LS	CD4bs	HIV-negative healthy adults: 5, 20, or 40 mg/kg, i.v. (0w) 5 mg/kg, s.c. (0w) 20, and 40 mg/kg, i.v. (0w, 12w, 24w) 5 mg/kg, s.c. (0w, 12w, 24w)	Half-life: 38 ± 12 days (i.v.) 33 ± 8.9 days (s.c.)
PGT121	V3 glycan	HIV-negative healthy adults: 3, 10, or 30 mg/kg, i.v. (0w) 3 mg/kg, s.c. (0w) On viremic subjects: 30 mg/kg, i.v. (0w) bNAbs during ATI: 3, 10, or 30 mg/kg, i.v. (0w)	Half-life: 23.5 days (HIV-) 19 days (HIV+, ART) 13 days (HIV+, Viremic) 30 mg/kg: 1.7 log10 drop in 5/9

w: week; m: month; i.v.: intravenous; s.c.: subcutaneous.

Phase I studies showed VRC01 to be safe and well tolerated without dose-associated toxicity or serious adverse effects in healthy adults and HIV-exposed infants [6, 25]. Its long-term clinical and functional activities have also been examined in healthy humans. Postinjection serum VRC01, administered intravenously (IV) (10-40 mg/kg) every 8 weeks or subcutaneously (SC) (5 mg/kg) every 2 weeks, was found to neutralize the majority of tested HIV strains, avidly capture HIV virions and mediate antibody-dependent cellular phagocytosis [26]. Another trial in HIV-1-infected patients revealed that a single dose of VRC01 was able to reduce plasma viraemia 1.1–1.8 log_10_ in six of eight ART-naïve subjects; the other two subjects with inadequate responses were found to have predominantly resistant viruses at baseline [27]. The terminal half-life (t_1/2_) of VRC01 in HIV-1-infected individuals was 12 days for intravenously administered infusions and 11 days for subcutaneously administered infusions, slightly lower than that in healthy adults. Further trials evaluated the effect of VRC01 on viral rebound after treatment interruption, and the results showed a median time to rebound of 4 weeks in the A5340 trial and 5.6 weeks in the National Institutes for Health (NIH) trial, constituting a small delay compared with historical controls [28]. However, VRC01 did not significantly prolong viral suppression in participants initiating ART during acute HIV-1 infection [29]. Passive administration of VRC01 is now being evaluated in high-risk individuals for protective efficacy against HIV-1 vertical and horizontal transmission (NCT02568215 and NCT02716675).

In the first clinical trial of 3BNC117, 12 uninfected and 17 HIV-1-infected subjects received a single intravenous dose of either 1, 3, 10 or 30 mg/kg [30]. The results showed that 3BNC117 was generally safe and well tolerated at all doses tested in both groups. In addition, clearance of serum 3BNC117 was faster in the HIV-1-infected group, with an average t_1/2_ of approximately 9 days and approximately 17 days in uninfected individuals. Antiviral effects were associated with the antibody dose, and upon receiving 30 mg/kg of 3BNC117, all 8 individuals displayed rapid and significant decreases in viral loads, with a median time of 7 days to reach the lowest level of viraemia; the mean drop in viral load was 1.48 log_10_ with durable activity for 4 weeks. In addition, 3BNC117 is able to enhance host antibody responses to heterologous tier 2 viruses in HIV-1-infected patients, irrespective of the initial neutralization potency and breadth and accelerated clearance of infected cells [7, 9]. 3BNC117 was then prompted into a phase IIa clinical trial to assess its efficacy in 13 HIV-1-infected individuals during analytical treatment interruption [31]. The results suggested that two (3 weeks apart) or four (2 weeks apart) infusions of 30 mg/kg 3BNC117 suppressed viral rebound for an average of 6.7 and 9.9 weeks, respectively, compared with 2.6 weeks for historical controls.

After these two CD4bs bNAbs, an antibody targeting a nonoverlapping epitope, 10-1074, was tested in humans [32]. In the clinical trial of 10-1074, 14 uninfected and 19 HIV-1-infected (3 on ART, 16 off ART) individuals received a single intravenous infusion at doses of 3, 10 or 30 mg/kg. Similar to VRC01 and 3BNC117, 10–1074 was well tolerated and cleared faster in HIV-1-infected participants than in seronegative subjects, with a half-life of 12.8 and 24.0 days, respectively. Individuals who received the dose of 10 mg/kg showed a rapid decline in plasma viral load of 1.08-1.56 log_10_ copies/ml, with a nadir at 7–9 days and a return to baseline levels within 3–4 weeks after administration. Participants with sensitive strains receiving 30 mg/kg of the antibody displayed a rapid decrease in viraemia, by an average of 1.52 log_10_ copies/ml, and the nadir was reached at a mean of 10.3 days after the infusion.

Recently, 3BNC117 and 10–1074 were administered in combination during analytical treatment interruption in a phase 1b clinical trial [33]. Eleven patients received three infusions of 30 mg/kg of each antibody every three weeks. Two individuals harbouring 10-1074- or 3BNC117-resistant viruses rebounded early; in contrast, individuals with antibody-sensitive viral reservoirs maintained suppression for a median of 21 weeks, and none of them developed resistance to either antibody. Furthermore, the combination of 3BNC117 and 10–1074 was administered to seven HIV-1 individuals with viraemia: one participant showed no response, two early rebound and four late rebound [34]. In those with dual antibody-sensitive viruses, immunotherapy resulted in an average reduction in HIV-1 viral load of 2.05 log_10_ copies/ml following one or three infusions, and none developed resistance to both antibodies.

Safety, pharmacokinetics and antiviral activity assessments in HIV-uninfected and HIV-infected adults have also been completed for another V3 glycan-targeting antibody, PGT121. PGT121 was found to be safe and well-tolerated, without related moderate and severe adverse events. The elimination half-life of PGT121 was 23.5 and 13 days in healthy and viraemic individuals, respectively. In viraemic individuals with high viral load (3.3-5.0 log_10_ copies/ml), PGT121 reduced plasma viral levels of 1.7 (1.3-2.1) log_10_ in 5/9 participants. These responders had PGT121-sensitive viruses at baseline but rebounded at 21–28 days with emergence of resistant mutations [35].

VRC01 was later modified as VRC01LS (a substitution of Met428Leu and Asn434Ser) to extend the antibody’s serum persistence. VRC01LS displayed a favourable safety in HIV-negative healthy adults, and the results revealed that its serum half-life was 71 ± 18 days after intravenous administration, more than 4-fold greater than wild-type VRC01 [36]. Additionally, an engineered version of VRC07, VRC07-523LS, has been demonstrated to be safe and shown improved pharmacokinetic and neutralization properties relative to VRC01 and VRC01LS in a phase 1 clinical trial [37]. VRC07-523LS administered alone or concurrently with other bNAbs, including 10E8VLS, PGT121, 10–1074 and PGDM1400, has also been advanced to additional clinical trials (NCT03387150, NCT03735849, NCT02256631, NCT02840474, NCT03565315, NCT03721510, and NCT03928821). The antibody N6LS and 10E8.4/iMab bispecific antibody are also being evaluated in humans (NCT03538626, NCT03875209).

## Challenges of bNAbs application

Despite the efficacy in reducing viraemia and maintaining viral suppression, bNAbs have not been found to be fully therapeutic in clinical trials. Challenges to the clinical utility of bNAbs include transient suppression of viraemia even at the highest doses (30–40 mg/kg), frequent emergence of resistance in rebound viraemia, suboptimal efficacy in cell-to-cell viral transmission, and unclear effects on the cell-associated HIV-1 reservoir. A better understanding of the challenges will help to improve bNAbs for HIV-1 prevention, therapy and cure.

### Transient suppression of viraemia

Durable suppression of HIV-1 replication is the goal of therapeutic treatment. Long-acting agents decrease the administration frequency and tend to reduce adverse effects. The short half-life of recently used small-molecule drugs requires daily therapy. The duration of viraemic suppression of bNAbs has been evaluated in human trials. As Lynch et al. reported, after infusion with VRC01, the plasma virus load transiently declined and rebounded to the baseline level during the observation period of 56 days [27]. In individuals receiving a single infusion of 3BNC117, 4 of the 8 participants returned to day 0 pre-infusion levels within 56 days [30]. During analytic treatment interruption, multiple doses of VRC01 did not provide durable viraemia suppression (less than 8 weeks) [28]. One factor leading to transiency is the insufficient potency of bNAbs. For example, VRC01 decreases viraemia to undetectable levels in subjects with low plasma viral loads (<1,000 copies/ml) at baseline, whereas viraemia is not fully suppressed among individuals with viral loads between 3,000 and 30,000 copies/ml.

The suboptimal half-life of bNAbs is another important factor. In a proportion of participants, plasma viraemia only rebounded at low antibody concentrations, which was associated with antibody decay. According to the current studies in humans, the average t_1/2_ of bNAbs is approximately 10 days in HIV-1-infected individuals and much shorter than that in uninfected individuals. The higher rate of antibody elimination in the presence of HIV-1 might be due to elevated levels of immunoglobulins and accelerated clearance of antigen–antibody complexes. Previous studies of anticancer antibodies have reported similar antigen-dependent increased clearance [38].

### Viral resistance

The increase in clinical resistance poses a considerable challenge to the use of antiretroviral drugs as therapeutic agents for HIV infection. Similar to small-molecule drugs, antibodies tend to select less-sensitive variants and/or promote resistance mutations. Sensitivity screening revealed that 67% of the individuals tested were sensitive to 3BNC117 and 58% to 10–1074 [34]. A low response or unresponsiveness has been observed in some individuals with viraemia, and studies have shown that these patients carry relatively resistant viruses as dominant populations of the preinfusion virus pool [27, 28]. Even in subjects with relatively sensitive variants, viral load rebound occurs in the presence of a bNAb. Virologic analysis based on 50% inhibitory concentration (IC_50_) and IC_80_ has demonstrated significantly increased bNAb resistance at postinfusion, suggesting bNAb-mediated selective pressure on the rebounding virus [27–30]. Postinfusion reduction in bNAb sensitivity results from the selection of pre-existing resistant variants or the development of resistance mutations. Lynch reported that all residues selected for in VRC01 postinfusion were present in preinfusion sequences [27]. However, participants carrying only sensitive virus were observed to undergo rebound with resistant virus. In addition, Caskey reported the emergence of multiple 10-1074-resistant viruses in the first weeks after infusion [32]. Loss of potential N-linked glycosylation sites (PNGSs) at position N332 or ^324^G(D/N)IR^327^ mutation was found in most of the sequences in the samples of week 4, and the rate of potential pre-existing resistant viral variants in circulation was probably lower than 1.0% [32].

A previous study showed that pre-existing viral sensitivity to bNAb does not predict the time of viral rebound [31]. In individuals carrying viruses that are highly sensitive to 3BNC117, as measured by the IC_50_, 100% neutralization was not achieved against preinfusion or rebound viruses, even at high antibody concentrations. This may suggest that IC_50_ or IC_80_ is not optimal for representing the virologic neutralization sensitivity prolife to bNAbs in individuals with viraemia. Neutralization of 50% or 80% most likely misses low-frequency resistant viruses and leads to rebound with resistant virus.

### Suboptimal efficacy in virus cell-to-cell transmission

Studies have suggested that HIV infects target cells via two mechanisms: cell-free viral particles or cell-to-cell transmission [39, 40]. Cell-to-cell transmission, at least in vitro, is up to approximately 3 orders of magnitude more efficient than free virus spread [41, 42]. Indeed, the cell-to-cell spread of HIV-1 appears to be primarily mediated through virological synapses involving HIV-1 Env-CD4 co-receptor interactions and adhesion molecules, similar to the immunological synapse [43, 44]. The formation of virological synapses may allow multiple infections of target cells and viral infections without exposure to an external environment, leading to rapid and efficient HIV-1 spread [45, 46]. Therefore, targeting and interfering with cell-to-cell transmission may reveal an effective and durable therapeutic or prophylactic agent. Several studies have shown that cell-mediated HIV-1 dissemination is less sensitive to antiretroviral drugs than cell-free viral infection [47–49]. The antiviral activity of bNAbs in cell-to-cell transmission has also been assessed.

Earlier studies attempting to determine the efficacy of bNAbs in inhibiting cell-to-cell transmission reached different and even conflicting conclusions due to the wide range of assay systems [50–52]. Recent studies have suggested that the capacity of bNAbs in cell-to-cell transmission varies depending on their mode of action and virus strains [10, 53]. A range of bNAbs and virus tests revealed decreased inhibitory activity against HIV-1 cell-to-cell transmission compared with cell-free transmission, and potent capacity in neutralizing free virus spread cannot predict equally relevant for inhibiting cell-to-cell transmission [53, 54]. More recently, Parsons examined the efficacy of bNAbs PGT121 against cell-associated SHIV infection and reported partial prevention of infection after cell-associated viral challenge [55]. These findings indicate a suboptimal efficacy of bNAbs against cell-to-cell transmission of HIV-1.

### Uncertain effect on the HIV-1 reservoir

The HIV-1 latent reservoir is the barrier to curing HIV-1 infections. This reservoir is likely established early during acute infection of the host and persists for life [56]. The quiescent integrated DNA is insensitive to antiretroviral therapy, leading to lifelong infection and viral rebound in most cases when ART is interrupted. Regardless, whether bNAbs will be able to target the latent reservoir and clear virally infected cells as potential immunotherapeutics remains a central question yet to be answered. In contrast to small-molecule drugs, antibodies potentially exert functions via Fc-mediated mechanisms such as antibody-dependent cellular cytotoxicity (ADCC) or antibody-dependent cell-mediated phagocytosis (ADCP). Such an immune opsonization enhances the clearance of target cells. In addition, immune complexes can activate dendritic cells, resulting in increased antigen processing and presentation to T cells, and activated T cells can directly kill target cells or act as helper cells for antibody responses [57].

In phase I clinical trials, bNAbs were found to engage host humoral immunity to HIV-1 and accelerate the clearance of HIV-1-infected cells. For instance, Schoofs reported that 3BNC117 infusion significantly enhanced host immunity to heterologous tier 2 HIV-1 viruses in nearly all study participants, irrespective of the initial neutralization potency and breadth [7]. In a humanized mouse model, 3BNC117 recognized CD4^+^ T cells infected with primary HIV-1 isolates and rapidly reduced the percentage of infected cells by Fcg receptor engagement [9]. However, Lynch reported that VRC01 infusion did not decrease the levels of cell-associated HIV DNA in ART-treated patients or subjects with viraemia, and no difference in the frequency of infected cells in the peripheral blood after two infusions of VRC01 was noted [27]. As humanized mice cannot fully recapitulate the human host and a relatively short time frame of bNAb therapy and follow-up in clinical trials, neither of the results is sufficient to determine the effect of bNAbs on the HIV-1 reservoir. Therefore, further studies are needed to determine whether bNAbs target the persistent reservoir or whether different bNAbs or engineered bNAbs will be more effective.

## Opportunities for increasing efficacy

**Figure 2.**
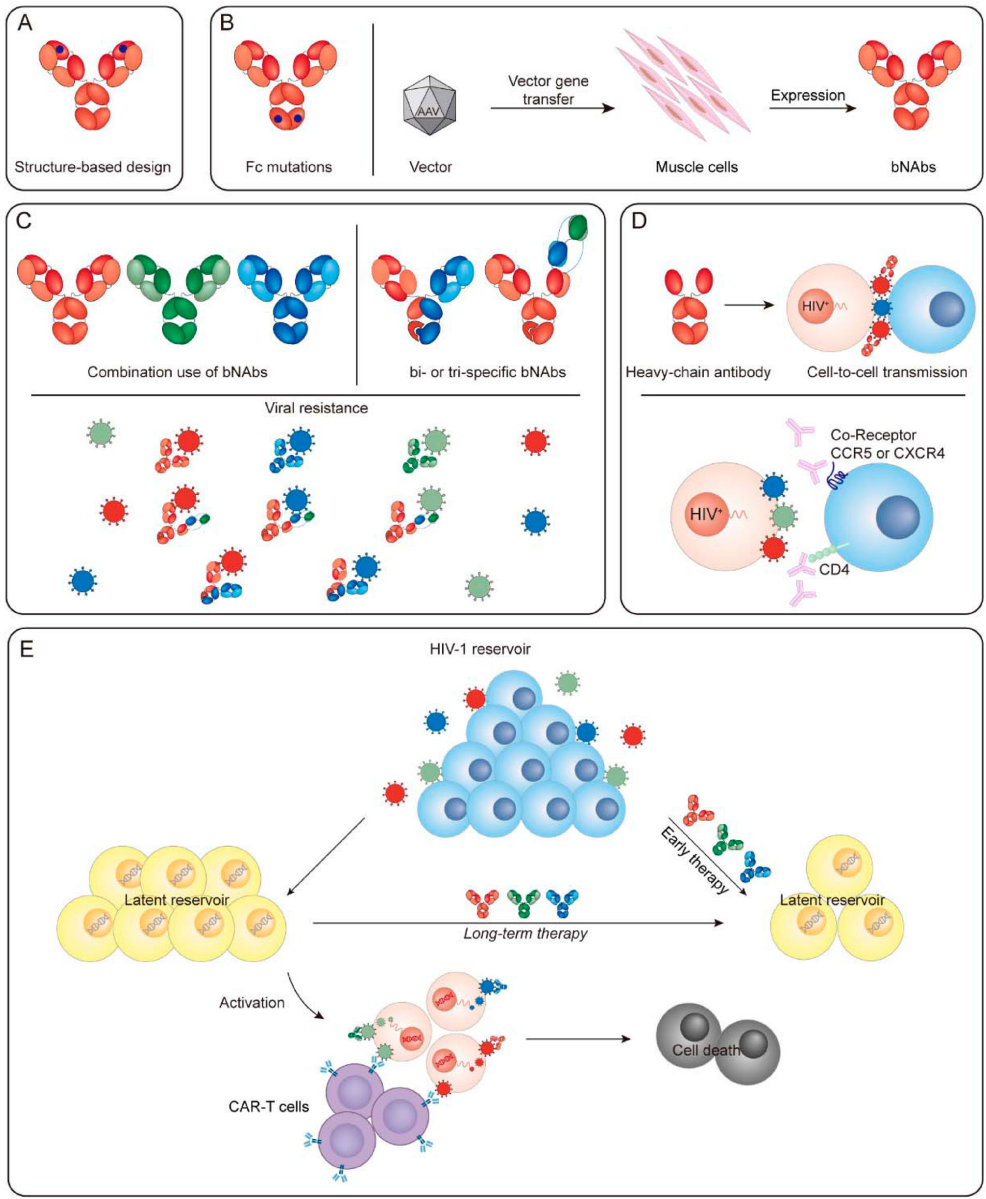
Strategies to increase efficacy of bNAbs. (A) Structure-based design to improve the potency or breadth of bNAbs. (B) Fc mutations and gene therapy to increase half-Life. (C) Combined use of bNAbs and creating multi-specific bNAbs to inhibit viral resistance. (D) Heavy chain antibody to access virus in cell-to-cell transmission, and antibody targeting CD4 or coreceptors to inhibit cell-to-cell spread. (E) Early therapy or long-term use of bNAbs and CAR-T in conjunction with latency reversal agents to affect HIV-1 reservoir.

In the face of all of these challenges, certain strategies are promising. Increasing the potency and half-life of bNAbs may result in durable viraemic suppression. Additionally, the combined use of bNAbs targeting nonoverlapping epitopes is beneficial for complete viraemic control and suppressing viral rebound followed by selection of resistant variants. Promoting access to the virological synapse probably inhibits cell-to-cell transmission. Early and prolonged treatments may affect the cell-associated HIV-1 reservoir (Figure 2).

### Increasing potency of bNAbs

The neutralization potency of parent bNAbs may be enhanced by rational optimization. NIH45-46G54W, a single substitution in CDRH2 by structure-based design, results in increased contact with the gp120 inner domain/bridging sheet as well as enhanced potency and breadth by an order of magnitude [58]. In addition, by increasing positive charges at the paratope surface of 10E8, the S30R/N52R/S67R triple substitution enhanced electrostatic interaction between the antibody and lipid bilayer, enabling 10E8 to bind to Env spikes with a higher affinity and neutralizing the virus with greater potency [59]. More recently, an FR3-loop grafting strategy was used to construct chimeric antibodies by engrafting the extended FR3 loop of VRC03 onto different bNAbs. Chimeric VRC01 FR3-03, VRC07-523-LS FR3-03 and N6 FR3-03 established quaternary contacts, with increased binding affinity and neutralization potency compared to the original antibodies [60]. Furthermore, the strategic placement of a glycan in the variable region of a monoclonal antibody can be used to improve its activity. The ibalizumab variant LM52 neutralized all HIV-1 strains tested at a potency more than tenfold higher than parental ibalizumab [61].

### Increasing the half-life of bNAbs

A longer half-life of bNAbs should maintain protective concentrations and durable HIV-1 suppression. However, the serum half-life is closely linked to the degree to which immunoglobulin G (IgG) binds to the neonatal Fc receptor for IgG (FcRn) [62]. One feasible method for increasing the half-life of an antibody is to modify the IgG crystallizable fragment (Fc) domain. The availability of newly engineered variants has greatly extended the duration of virus suppression following a single dose of bNAb. VRC01-LS, a site-directed mutation derivative of VRC01, had a threefold longer serum half-life than VRC01 in nonhuman primates. This modification not only prolonged the serum half-life but also increased the persistence of antibodies at mucosal sites, which enhanced mucosal immunity [63]. Moreover, introduction of this modification into VRC01 reportedly increased the median protection against repeated SHIV challenges after a single injection [23], and a recent clinical trial confirmed that the half-life of VRC01-LS was 4-fold longer than that of unmodified VRC01 and that VRC01-LS could maintain viraemia suppression for 6–12 months in individuals with sensitive viruses [36]. Regarding other bNAbs, LS substitution in 3BNC117 and 10–1074 leads to a significant increase in half-life and protective efficacy against SHIV infection [64]. Therefore, half-life extension might allow for increasing the duration of viraemia suppression and achieving therapeutic levels with reduced frequency.

As an alternative strategy, vector-mediated gene transfer can provide sustained expression of antibodies and significantly extend the lifetime of bNAbs. In animal models, gene-delivered antibodies generated long-term neutralizing activity and protected monkeys and humanized mice from intravenous and mucosal SIV/HIV infection [65–68]. However, obstacles to widespread use of such an approach in humans include limited carrying capacity and anti-transgene responses [69]. Therefore, improved gene expression cassettes, alternative vectors and potent bNAbs are needed to make this an effective strategy for durable antibody expression.

### Combination use of bNAbs

Similar to the development of antiretroviral drugs for HIV infection, a combination of bNAbs that target different sites on the HIV envelope glycoprotein are being considered for effective therapy and possibly prevention.

Preclinical studies have revealed that administration of a cocktail of bNAbs effectively suppressed HIV-1 viraemia for 60 days after the interruption of treatment [70]. Moreover, the combination of bNAbs significantly limited signature-resistant mutations. More recent research showed that the combination of PGT121 and PGDM1400 provided 100% protection against mixed SHIV challenge but that PGT121 or PGDM1400 alone failed to protect against mixed challenge [71]. These results suggest that single-bNAb-selected resistant viruses generate a diverse infection and that the combination of PGT121 and PGDM1400 display remarkable complementarity, as viruses resistant to one of these antibodies are generally sensitive to the other antibody.

Furthermore, clinical tests have revealed that postinfusion viruses resistant to a certain bNAbs exhibit no corresponding change in sensitivity to bNAbs targeting different and nonoverlapping epitopes. For example, VRC01-resistant variants showed no corresponding change in sensitivity to the MPER-targeting antibody 10E8 [27], and viral rebound during ATI with 3BNC117 did not well correlate with increased resistance to 10–1074 [30]. Moreover, no correlations between the emergence of resistance to 10–1074 and resistance to antibodies binding to independent sites on the HIV-1 envelope (VRC01, 3BNC117 and PGDM1400) were found [32]. Clinical trials have determined that the combination of 3BNC117 and 10–1074 significantly prolonged viral suppression and that it was relatively difficult for HIV-1 to develop resistance to both antibodies [33]. The variability of bNAbs reacting with divergent epitopes and their abilities to block both transmission modes and to distinctly neutralize pre- and post-CD4 attachment further highlights the need to create optimal combinations of bNAbs.

Finally, as alternatives to antibody combinations, bi- or tri-specific anti-HIV-1 antibodies, such as VRC07-PG9-16, 10E8.2/iMab, VRC01/PGDM1400-10E8v4, and 10E8v4/PGT121-VRC01, demonstrate vastly increased antiviral activity, and clinical trials are ongoing or about to be initiated [72–75]. Overall, combinations of antibodies targeting nonoverlapping epitopes display remarkable complementarity and may dampen the development of viral resistance and lower doses.

### Evaluating cell-to-cell transmission

High inhibition efficacy against free virus is associated with high neutralization activity prior to CD4 engagement. Nevertheless, the effect of bNAbs on targeting the viral envelope post-CD4 engagement appears to be effective during cell-to-cell transmission, as cell-to-cell transmission is mediated through the formation of the virological synapse (VS). This effect may explain why some bNAbs with great potency in vitro lack comparable activity in vivo and fail to suppress viral load to undetectable levels in humans in the long term. Thus, inhibitory activity in cell-to-cell transmission must be considered when determining bNAb candidates for further study.

The activity of bNAb-mediated inhibition of HIV-1 cell-to-cell transmission is likely impacted by steric hindrance at the virological synapse. Therefore, antibodies with smaller sizes may gain access to the VS and increase inhibitory activity. It has been reported that the Fab fragment of 10E8 displays more comparable neutralization potency during cell-free and cell-to-cell transmission than the full-size 10E8 at the macrophage-to-T cell [76]. In addition, a llama antibody J3 with a heavy chain-only variable region (VHH) is a potent inhibitor of HIV-1 cell-to-cell spread. Notably, the full-length heavy chain-reconstituted VHH (J3-Fc) effectively neutralizes cell-to-cell spread, suggesting that small size is not the only determinant of potency and that other factors are worthy of further exploration [77]. Additionally, due to the higher multiplicity of infection (MOI) during cell-to-cell transmission, maintaining high concentrations of bNAbs is crucial for protecting against cell-associated viruses [54]. Alternatively, mAbs that target CD4 or coreceptors competitively or uncompetitively bind to CD4/CCR5 with higher affinity than to HIV glycoprotein 120 (gp120) and might inhibit viral entry as well as cell-to-cell spread [78–80].

Overall, a better understanding of the mechanisms of cell-to-cell transmission in vivo may promote the use of bNAbs for effectively inhibiting this mode of HIV-1 spread. Indeed, defining the relative contribution of cell-free and cell-to-cell virus transmission in vivo is a priority. Mathematical analysis has revealed that cell-to-cell transmission is more likely to lead to escape mutants [52], highlighting the significance of controlling virus replication through the cell-to-cell transmission pathway. In addition, differentiating virological synapses from immunological synapses and identifying viral particles produced at the virological synapse will be beneficial for specific and accurate therapies.

### Affecting the HIV-1 reservoir

Initiation of cART during the early stage of HIV-1 infection accelerates the decay of infected CD4^+^ T cells, leading to significantly lower levels of cell-associated HIV-1 DNA after long-term therapy [81]. The results suggest that early antiretroviral therapy may affect the seeding of viruses and limit the size of the latent viral reservoir. Similarly, early immunotherapy can clear viral foci and thereby protect against the establishment of viral reservoirs. In a preclinical trial, infant rhesus macaques received subcutaneous injection of bNAbs on days 1, 4, 7 and 10 after virus challenge. The results showed that viruses in the blood and tissues were killed in all bNAb-treated rhesus macaques at 6 months after exposure; moreover, no anti-SHIV T cell responses in blood or tissues were detected at necropsy, and no viruses emerged after CD8^+^ T cell depletion [82]. In a recent study [83], animals received a single 2-week course of combination bNAb therapy with 3BNC117 and 10–1074 at three days after infection; these animals displayed virus control for 56–177 days, but those treated with cART experienced virus rebound after interruption. Among 13 bNAb-treated rhesus macaques, the proportion of replication-competent virus-carrying cells was less than 1 per 10^6^ circulating CD4^+^ T cells in 6 controllers, and four additional rhesus macaques maintained CD4^+^ T cells and very low levels of viral load for over 2 years. The results also indicated that in contrast to cART, bNAb therapy during acute SHIV infection promotes the emergence of potent CD8^+^ T cell immunity that is able to persistently suppress virus replication. This mechanism of immunotherapy may constrain the establishment and maintenance of viral reservoirs and control infection in humans.

Another possible way of impacting the HIV-1 reservoir is to prolong treatment with bNAbs. In recent clinical trials, one or more doses of bNAb were administered, and individuals were observed for 2–5 months [27]. As it is possible that the therapeutic strategy or time frame was not sufficient to affect the latent viral reservoir, prolonging treatment with bNAbs and extending the follow-up time frame may lead to reservoir depletion. Additionally, the administration of engineered antibodies with improved Fc-mediated cell killing, such as ADCC and complement-dependent cytotoxicity (CDC), may reduce the latent viral reservoir [9].

The latent HIV-1 reservoir is in a quiescent state and may not express viral antigens recognized by antibodies. In this regard, bNAbs in combination with latency-reversal agents that activate virus expression on the cell surface may potentiate the ability of bNAbs to kill infected cells. It has been reported that the combination of inducers and bNAbs interferes with the establishment and maintenance of the HIV-1 reservoir in a humanized-mouse model [84]. Additionally, a recent study showed that bNAb PGT121 together with a Toll-like receptor 7 (TLR7) agonist delayed viral rebound in SHIV-infected monkeys, probably due to a reduction in the viral reservoir [85]. Furthermore, chimeric antigen receptor (CAR)-T cells, with the extracellular domain from the single-chain variable fragment of bNAb, can effectively kill reactivated HIV-1-infected CD4^+^ T cells [86]. Multispecific anti-HIV duoCAR-T cells display potent elimination of HIV-infected cells and mitigate CD4^+^ T cell loss in a humanized mouse model of intrasplenic HIV infection [87]. A newly designed *convertible*CAR-T cell system capable of binding to a variety of bNAbs yields greater breadth and control, representing a potential strategy for targeting the latent HIV-1 reservoir [88].

## Conclusion

Given the difficulties in developing HIV-1 vaccines and eliminating an established infection, passive transfer of monoclonal antibodies is a promising strategy for HIV-1 prevention and therapy. Despite the cost advantage of the currently used small-molecule drugs, bNAbs have longer half-lives and the potential to kill HIV-1-infected cells and affect the course of HIV-1 infection by directly engaging host immunity. Currently, bNAbs are advancing into clinical stages in succession and display efficacy in suppressing viraemia and in controlling viral rebound. Despite the overall challenges, possible strategies and opportunities exist simultaneously.

According to the results of recent human trials, a better understanding of the roles that antibody concentration, virus selection, and preinfusion viral levels play in the rebound of virus load to baseline is required to achieve full therapy with bNAbs. In addition, the 100% neutralization capacity of bNAbs or maximum percentage of inhibition (MPI) may need to be considered. Furthermore, prescreening for resistance appears to be necessary, though it is not an easy task. Finally, considering the numerous diversity and high variability of HIV-1, the isolation and modification of additional potent bNAbs targeting independent HIV-1 envelope epitopes, and the development of optimal antibody cocktail formulations are required to expand the future use of immunotherapy regimens.
